# Organically Modified Silica with Pyrazole-3-carbaldehyde as a New Sorbent for Solid-Liquid Extraction of Heavy Metals

**DOI:** 10.3390/molecules19010247

**Published:** 2013-12-24

**Authors:** Smaail Radi, Said Tighadouini, Maryse Bacquet, Stéphanie Degoutin, Francine Cazier, Mustapha Zaghrioui, Yahia N. Mabkhot

**Affiliations:** 1 Laboratoire de Chimie Appliquée et Environnement (LCAE), Faculté des Sciences, Université Mohamed I, Oujda 60 000, Morocco; 2 Centre de l’Oriental des Sciences et Technologies de l’Eau (COSTE), Université Med I, Oujda 60 000, Morocco; 3 Unité Matériaux et Transformations UMR8207 (UMET), Equipe Ingénierie des Systèmes Polymères, Université des Sciences et Technologies de Lille, Bâtiment C6 salle 119-59655 Villeneuve d’Ascq, France; 4 Université Lille Nord de France, F-59000 Lille, Unité de Chimie Environnementale et Interactions sur le Vivant, 145 Avenue M. Schuman F-59140 Dunkerque, France; 5 Laboratoire GREMAN CNRS-UMR 7347 IUT de BLOIS, Université François-Rabelais de Tours, 15 rue de la Chocolatrie 41029 Blois, France; 6 Department of Chemistry, Faculty of Science, King Saud University, P.O. Box 2455, Riyadh 11451, Saudi Arabia

**Keywords:** chemically modified SiO_2_, synthesis, characterization, adsorption, Pb(II), Cd(II), Cu(II), Zn(II)

## Abstract

A new chelating matrix, SiNP, has been prepared by immobilizing 1.5-dimethyl-1*H*-pyrazole-3-carbaldehyde on silica gel modified with 3-aminopropyl-trimethoxysilane. This new chelating material was well characterized by elemental analysis, FT-IR spectroscopy, cross polarization magic angle spinning solid state ^13^C-NMR, nitrogen adsorption-desorption isotherm, BET surface area, BJH pore size, and scanning electron microscopy (SEM). The new product exhibits good chemical and thermal stability as determined by thermogravimetry curves (TGA). The new prepared material was used as an adsorbent for the solid-phase extraction (SPE) of Pb(II), Cd(II), Cu(II) and Zn(II) from aqueous solutions using a batch method, prior to their determination by flame atomic adsorption spectrometry. The adsorption capacity was investigated using kinetics and pH effects. Common coexisting ions did not interfere with separation and determination.

## 1. Introduction

Environment pollution by heavy metals has caused lately much concern because of their general and specific toxicities. The most toxic heavy metals, namely lead, cadmium, copper and zinc, can be distinguished from other pollutants, because they cannot be degraded naturally, but rather accumulate in living organisms. Therefore they cause different diseases and disorders, even at low concentrations [1,2,3,4,5,6]. Therefore, determination of heavy metals in environmental and biological materials is an important screening procedure in environmental pollution and occupational exposure studies.

Traditionally, extraction is carried out liquid-liquid extraction, co-precipitation, and ion exchange, *etc.* These methods have non-economic disadvantages. They often require large amount of high purity organic solvents, some of which are themselves harmful to health and cause environmental problems. Nowadays, several methods are used for pretreatment of the samples. Solid phase extraction (SPE) [7,8,9,10,11,12], has commonly been used as a technique for pre-concentration/separation of various inorganic and organic species. SPE has several major advantages that include higher enrichment factors, simple operation, safety with respect to hazardous samples, high selectivity, lower cost and less time, the ability to combine it with different modern detection techniques [13]. 

A variety of ligands or functional groups are immobilized onto a solid support matrix as a solid phase extractant for the purpose of extraction and enrichment of trace metal ions from environmental samples. Silica gel is of great importance as a solid support because it possesses some definite advantages [14]. The silica support is chosen for its high surface area, high mechanical and thermal stability. In addition, it is easily modified [15], by reacting with organofuctionalized silanes through its surface silanol groups. These covalently bonded organic groups are highly stable and resistant to removal from the surface by organic solvents or water [16]. To this end, a great number of organic molecules were immobilized on silica gel surface, xylenol orange [17], 2-thiophenecarboxaldehyde [18], di(*n*-propyl)thiuram disulfide [19], 4-acylpyrazolone [20], aminothioamidoanthraquinone [21], 1,8-dihydroxyanthraquinone [22], murexide [23], oxime derivatives [24], resacetophenone [6], diphenyldiketone monothiosemicarbazone [25]. These systems can be operated indefinitely without loss of the expensive organic molecules. Their potential applications are attributable essentially to the nature of the grafted ligands. Indeed, the most commonly attached molecules have chelating ability due to their donor atoms, such as oxygen, nitrogen and sulphur, which have a large capability to form complexes with a series of metal ions, leading in some cases, to distinguishable selective extraction properties. 

In this context, for many years, the ability of pyrazole and its derivatives to act as ligands with sp^2^ hybrid nitrogen donors have been the research subjects of many coordination chemists. This is evident from the large number of articles on this topic, several of them being reviews [26,27,28]. In continuation of our work in this field [29,30,31,32], this paper describes the synthesis and the characterization of a new material obtained by grafting onto porous silica functionalized compounds which can act as in a *N,N'*-bidentate fashion [33,34] forming five membered chelating rings. The immobilization of this ligand on silica gel was carried out with a long arm spacer in order to facilitate the contact between the receiver and the metal ion. The new chelating material was well characterized and its adsorption capacities towards highly toxic heavy metals ions such as Pb(II), Cd(II), Cu(II) and Zn(II) was investigated and the extracted amounts of metals ions were determined by atomic absorption measurements. This new material presents high adsorption of lead compared to the other tested metals ions.

## 2. Results and Discussion

### 2.1. Linker Synthesis

**Scheme 1:**
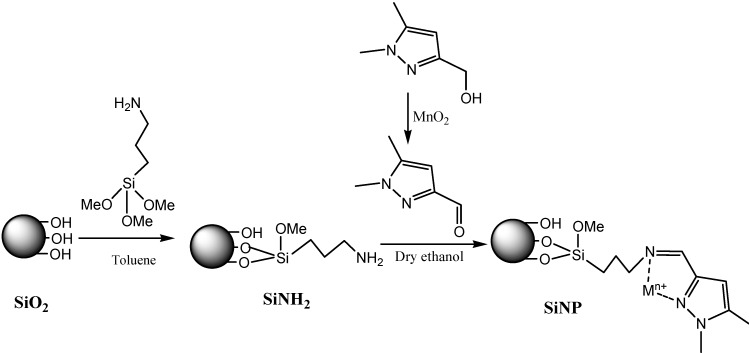
The synthesis route of modified chelating material.

The synthetic procedure for the new chelating material is summarized in Scheme 1. The preparation involves the reaction of activated silica gel with 3-aminopropyltrimethoxysilane in toluene to install amino groups attached to the silica surface [35]. These NH_2_-groups onto the silica surface were then reacted with 1,5-dimethyl-1*H*-pyrazole-3-carbaldehyde under mild conditions (reflux, atmospheric pressure and 8 h) using anhydrous ethanol as solvent, to form the new chelating sorbent SiNP.

### 2.2. Characterization

#### 2.2.1. Elemental Analysis

The elemental analysis of carbon and nitrogen (not present in the starting activated silica) of aminopropylsilica SiNH_2_ makes it possible to characterize and highlight the introduced organic group on the silica surface. The microanalysis results (%C = 4.46, %N = 1.66 and %H = 1.27) suggests that two methoxy groups were substituted by silanol. The final SiNP-Schiff base material showed also an increase in the percentage of C, N and H (%C = 5.32, %N = 1.90 and %H = 1.34), which means that the pyrazole unit was immobilized on the silica gel surface. 

#### 2.2.2. FT-IR Characterization

**Figure 1:**
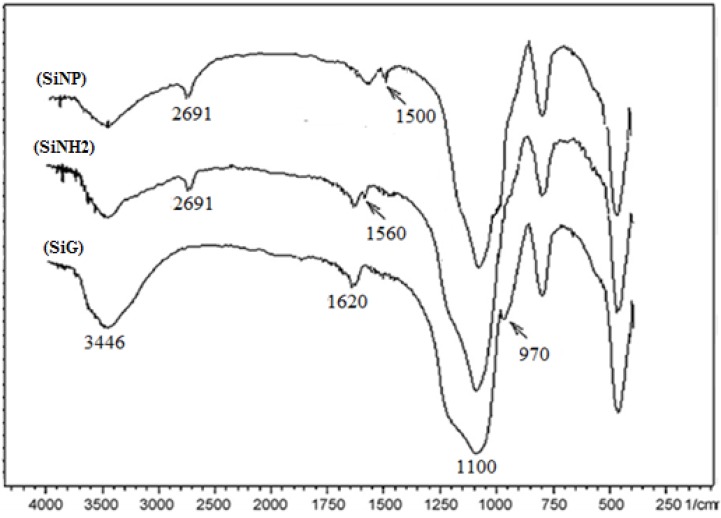
FT-IR Spectra of free silica (SiG), 3-aminopropylsilica (SiNH_2_) and (SiNP).

To confirm the presence of functional groups in the material, FT-IR spectra were performed for free silica gel, SiNH_2_ and SiNP materials (Figure 1). The sharp features around 1,100 cm^−1^ indicated Si-O-Si stretching vibrations. The presence of adsorption water was reflected by ν(OH) vibration around 3,446 and 1,620 cm^−1^. The bonds around 970 cm^−1^ resulted from Si-O vibration [36]. Compared to free silica gel, the spectrum of SiNH_2_ exhibits some new peaks such as the CH_2_ vibration band at 2,691 cm^−1^ and the NH_2_ vibration at 1,560 cm^−1^ [37,38]. The characteristic features of SiNP compared with SiNH_2_ were the disappearance of the adsorption band at 1,560 cm^−1^ due to the reaction of the primary amine (-NH_2_) and the appearance of a new characteristic bond around 1,500 cm^−1^ resulting from C=N and C=N vibrations, which confirms the anchoring of the organic molecule onto the silica surface.

#### 2.2.3. Scanning Electron Micrographs

**Figure 2:**
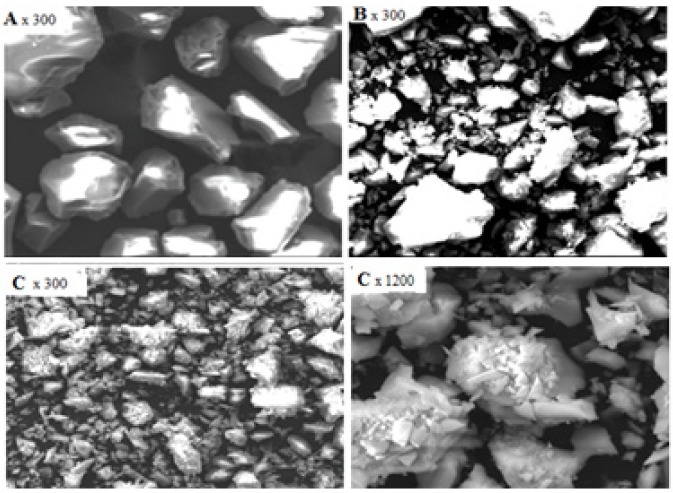
SEM images of free silica (**A**), SiNH_2_ (**B**) and SiNP (**C**).

Scanning electron micrographs (SEM) were obtained on the free silica and chemically modified silicas in order to detect differences in their surfaces. SEM of silica gel, SiNH_2_ and SiNP in Figure 2 were obtained at 300× and 1,200× magnification. The SEM was displayed to clarify the un-agglomeration of the silica gel particles after treatment to support the claiming of regular distribution of the functional group on the whole surface. It was evident that the loaded functional groups were distributed on the whole surface that made the surface of the product SiNP become rough.

#### 2.2.4.TGA Analysis and Thermal Stability

**Figure 3:**
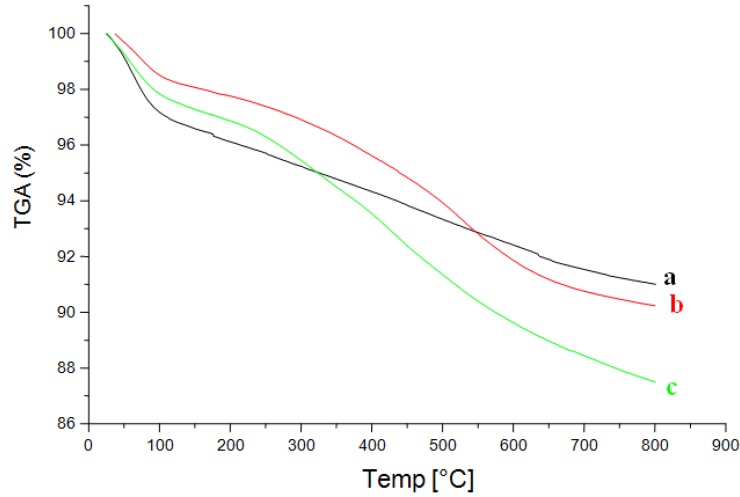
Thermogravimtric curves of free silica (**a**), SiNH_2_ (**b**) and SiNP (**c**).

The thermogravimetric curves for all surfaces enable the establishing of information on thermal stability and also to confirm the amount of the compounds immobilized, as shown in Figure 3. The profile indicates a degradation process between 146 and 800 °C which confirms the high thermal stability for the prepared material. 

The free silica presents a first mass loss stage of 3.15% in the interval from room temperature to 110 °C, assigned to physically adsorbed water and a second loss of 5.85% from 110 to 800 °C assigned to condensation of the free silanol groups which causes siloxane bond formation (Si-O-Si) [39,40]. Again two distinct mass loss steps were detected for the SiNH_2_ sample. The first one, a small mass loss of 1.56% in the room temperature to 100 °C range is attributed to the remaining silanol hydration water, as a consequence of the use of these groups in the immobilization process. On the other hand, a pronounced mass loss increase of 9.77% was observed for the second step, between 208 and 800 °C, which corresponds to the organic matter added onto the surface during immobilization. The final SiNP material presented two distinct mass loss stages. Following the preceding interpretation, the first mass loss of 2.27% in the 25–102 °C range is assigned to adsorbed water, and other mass loss of 12.49% between 231.47 and 800 °C is attributed to the decomposition of the pyrazole fraction immobilized on the surface of silica gel, together with the condensation of the remaining silanol groups. The pronounced increase in mass loss reflects the higher amount of the anchored organic groups.

#### 2.2.5. ^13^C-NMR Characterization

**Figure 4:**
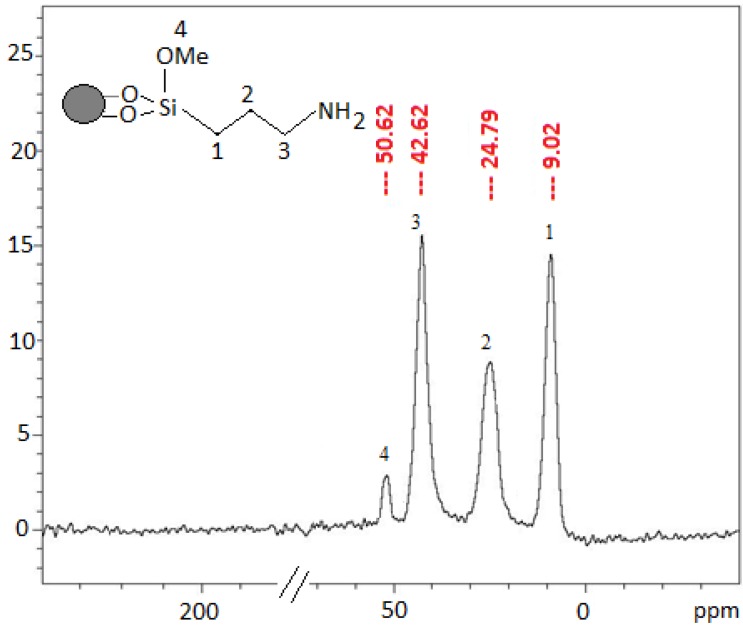
^13^C-NMR spectra of 3-aminopropylsilica (SiNH_2_).

Important features related to the immobilization of pendant groups on the inorganic structure of the formed hybrid can be obtained through solid state ^13^C-NMR spectroscopy, as shown in Figure 4. The signals observed for 3-aminopropyl-silica SiNH_2_ at δ = 9.02, 24.79 and 42.62 ppm have been assigned to the propyl carbon Si-CH_2_, -CH_2_- and N-CH_2_, respectively. The signal at 50.62 ppm was assigned to the unsubstituted methoxy group as confirmed by microanalysis.

#### 2.2.6. Chemical Stability

Chemical stability of the newly synthesized material SiNP was examined in various acidic and buffer solutions (pH 1–7). No change in the material structure was observed even after 24 h of contact. The high stability exhibited by the attached organofunctional group is presumably due to the pendant group. It has been shown that when the length of the hydrocarbon bridge was more than two methylene groups, the rupture of Si–C bond did not occur in a mineral acid medium, due to the length of the chain; longer chains no longer have a functional handle that can undergo β-elimination of the Si cation [41,42].

#### 2.2.7. Surface Properties

**Figure 5:**
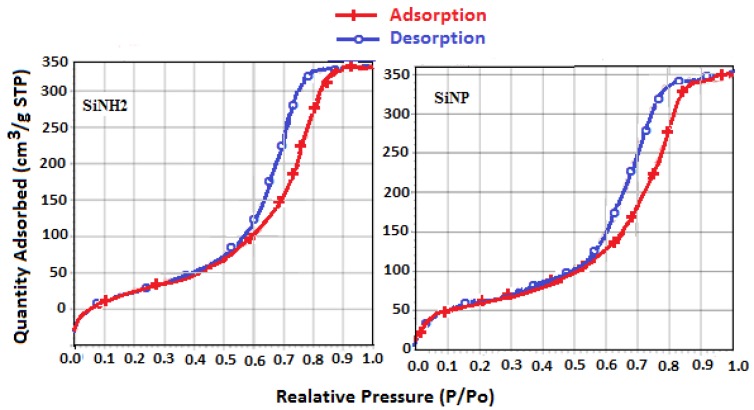
Nitrogen adsorption-desorption isotherm plots of SiNH_2_ and SiNP.

To show the porosity changes of the silica induced by the introduction of 3-aminopropyl and pyrazole unit, we measured the surface area S_BET_ (Brunauer–Emmett–Teller), pore volumes, and pore diameters of both silica and its derivatives with nitrogen adsorption–desorption isotherms (Figure 5) and by Barrett–Joyner–Halenda (BJH) pore diameters methods [43,44]. The density of the pendant groups covalently attached to the inorganic silica backbone changes the original characteristics of the surface. As shown in Table 1, the initial specific surface area S_BET_ of 305.21 m^2^g^−1^ and a pore volume of 0.77 cm^3^g^−1^, decreases as the immobilization takes place to give 283.08 m^2^g^−1^ and a pore volume of 0.69 cm^3^g^−1^. A decrease in S_BET_ is mainly due to the presence of the organic moieties that can block the access nitrogen to the silica base. On the other hand, we observed that SiNP has an additional BET surface area decrease as additional group immobilization takes place to give 236.60 m^2^g^−1^, and a pore volume of 0.64 cm^3^g^−1^. The decreased surface area and pore volume in SiNP are attributable to the grafted 1.5-dimethyl-1*H*-pyrazole-3-carbaldehyde.

**Table 1:** Physical properties of silica derivatives.

Silica derivatives	Specific surface S_BET_ (m^2^ g^−1^)	Pore volume (cm^3^ g^−1^)
Free silica	305.21	0.77
SiNH_2_	283.08	0.69
SiNP	236.60	0.64

Moreover, the nitrogen adsorption–desorption isotherm for silica derivatives, shown in Figure 5, are type IV according to the IUPAC classification and display a pronounced hysteresis for partial pressures P/P_0_ > 0.4.

### 2.3. Solid–Liquid Adsorption of Metal Ions by SiNP

The effects of pH and shaking time on the extraction of the three metal ions were studied by the batch method. The modified silica gel (10 mg) was equilibrated by shaking with 10 mL of a solution containing different concentrations of metal ions (243.01 mg/L for Pb(II), 102.93 mg/L for Cd(II), 75.63 mg/L for Cu(II) and 74.02 mg/L for Zn(II)), for different time intervals (1, 15, 30 min and 1, 1.5, 2, 3, 4, 5, 6, and 24 h) and different pH values (1–8). The metal ions were in excess over the sorption capacity. The concentration of metal ions was determined by means of atomic absorption measurements. The amount of metal ions adsorbed by the synthesized material SiNPz from aqueous solution was calculated using the following equations [45]:

Q_M_ = (C_0_ − Ce) × V / W

Q_W_ = Q_M_ × M

where Q_M_ is the amount of the metal ion on the adsorbent (mmol/g), Q_W_ is the amount of the metal ion on the adsorbent (mg/g), V is the volume of the aqueous solution (l), W is the weight of the adsorbent (g), C_0_ the initial concentration of metal ion (mmol/L), Ce the equilibrium metal ion concentration in solution (mmol/L) and M the atomic weight for metals (g/mol). Analyses were performed in duplicate for each sample and the mean data are reported.

#### 2.3.1. Effect of pH

It is well-known that binding of metal ions to the chelate compounds either in solution or loaded on solid supports is mainly dependent on several factors such as the nature, charge and size of the metal ions [46,47], nature of the donor atoms and their binding characteristics [48,49], and the buffering conditions. These factors are very well documented in solution chemistry as well in solid-phase extraction of certain metals by organic chelates immobilized on the surface of solid supports such as silica gel, nanomaterials or polymeric species. Therefore, to evaluate the suitability of the newly synthesized SiNP for metal ions extraction and binding, we studied the effect of pH of the metal ion solution on the metal capacity values as one of the most significant controlling factors in such a process. 

**Figure 6:**
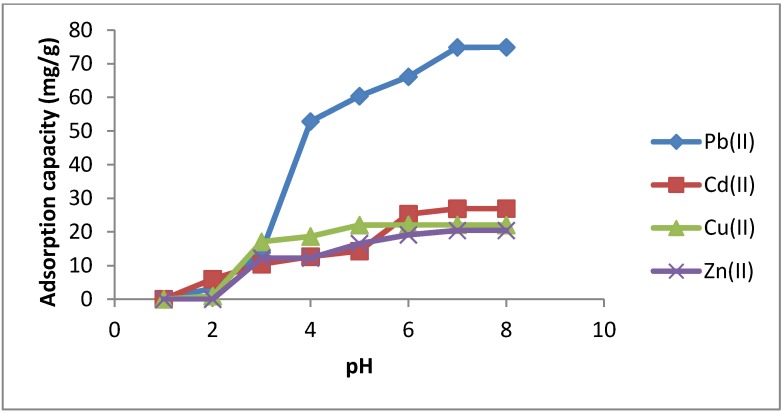
Adsorption kinetics of Pb(II), Cd(II), Cu(II) and Zn(II) on SiNP.

The adsorption properties of SiNP were investigated in the pH 1–8 range as shown in Figure 6. Results reveal that the metal ion uptake of the adsorbent varies significantly as the pH changes. At lower pH values, the retention of metal ions by the functionalized silica SiNP is not significant since the ligand must be almost entirely in its protonated form. As the pH increases, the protonation becomes weak, which enhances the chelation and adsorption of metal ions. At pH > 8, the retention of metal ions decreased because of the hydrolysis of metal ions (leading to the hydroxides of M(II): M(OH)^+^ and M(OH)_2_), this makes it difficult to distinguish between the hydrolyzed or adsorbed M(II). Therefore, the optimum pH for the maximum sorption of Cu(II) was at pH ≥ 5, Cd(II) and Zn(II) at pH ≥ 6 and Pb(II) at pH ≥ 7. Data are given in Table 2.

**Table 2:** Metal ion uptake of SiNP (Q_w,_ mg/g) according to pH.

pH	Pb(II)	Cd(II)	Cu(II)	Zn(II)
1	0	0	0	0
2	3.26	5.99	0.96	0
3	13.92	10.43	17.08	12.24
4	52.83	12.66	18.68	12.25
5	60.38	14.34	22.04	16.55
6	66.14	25.29	22.1	19.13
7	74.86	26.89	22.08	20.43
8	74.89	26.93	22.06	20.43

#### 2.3.2. Effect of Stirring Time

**Figure 7:**
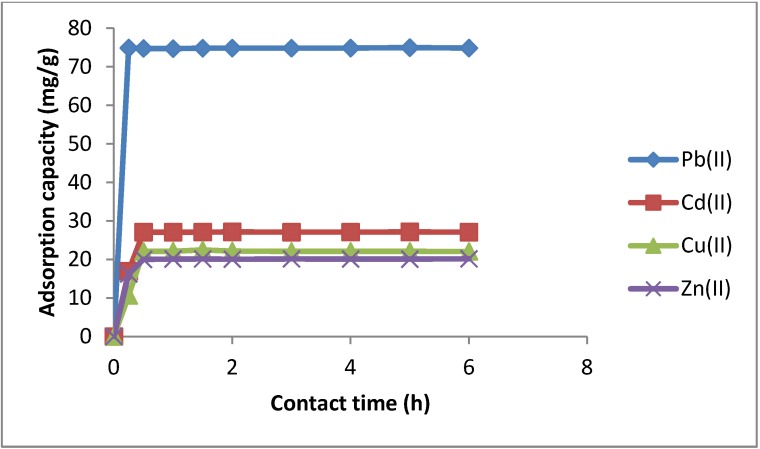
Effect of pH value on the retention of Pb(II), Cd(II), Cu(II) and Zn(II) on SiNP.

The stirring time used for the adsorption of the metal ion by the modified silica gel and the attainment of equilibrium conditions is of considerable importance. Effect of stirring time on the adsorption of Pb(II), Cd(II), Cu(II) and Zn(II) by SiNP was studied by batch experiments. As can be seen from Figure 7, the kinetic curves of Pb(II), Cd(II), Cu(II) and Zn(II) showed that the adsorption was rapid and the plateau was reached after about 30 min of contact. The rapid adsorption of different metal ions suggests that the two nitrogen active donor atoms on the modified silica gel surface are oriented in such a way that their accessibility is not hindered and consequently, fast interaction with the free metal ions present in solution is feasible. Indeed, the two nitrogens (of the grafted pyrazole and of the imine) act as a convergent chelating bidentate donor. The term convergent refers to the nitrogen donor atoms coordinating to the same metal center, thus leading to a five-membered ring which is part of several such rings when the whole ligand is considered. It is well known that five-membered ring chelates are more stable than six-membered and four-membered ones [50]. The rapid kinetics have a significant practical importance, as it will facilitate smaller reactor volumes ensuring efficiency and economy.

The variation in sorption capacities of various metal ions probably arises due to their size, degree of hydration, and binding constants of their complexes with the matrix.

#### 2.3.3. Effect of Coexisting Ions

It is well know that metal cations and acyclic pyrazolic compounds do not complex alkali metal cations at all, while the macrocyclic pyrazolic ligands form complexes both transition and alkali metals [51,52,53]. Thus, the effects of common coexisting ions in water samples on the recovery of each metal were also studied. In these experiments, 50 mL of a solution containing 0.1 μg/mL of a metal ion and various amounts of interfering ions were treated according to the recommended procedure. An ion was considered to interfere when its presence produced a variation in the extraction recovery of sample more than ±5%. The results show that in excess of 10,000-fold, the Li^+^, K^+^, Na^+^, Ca^2+^ and Mg^2+^ ions show no significant interferences in the extraction and determination of each Pb(II), Cd(II), Cu(II) and Zn(II) metals. As can be seen, SiNP has a high tolerance limit for alkali and alkaline earth metals. This is particularly useful for the analysis of transition elements of group 12 and Pb(II), in natural water samples, for example seawater, which contains large amounts of alkali and alkaline earth metal ions.

#### 2.3.4. Comparison with Alternative Materials

Table 3 shows the adsorption of Pb(II), by other material reported in the literature. It is clear that the functionalized silica described in this work presents further improvement and shows better values and higher affinity for the effective adsorption for Pb(II) and other metals under study. 

**Table 3:** Comparison of SiNP with other reported sorbents for Pb(II) absorption.

Support: silica gel/ligand	Reference	Capacity (mg of Pb^2+^/g of silica)
Pyrazol-3-ylimine (this work)	-	74.89
Gallic acid	[54]	12.63
Ethylediamine derivatives	[55]	38.12
C,N-pyridylpyrazole	[56]	09.5
Thiophene	[57]	11.3
Acid red 88	[58]	03.35
Dithizone	[59]	08.28
Resacetophenone	[6]	13.79
Tris(2-aminoethyl) amine	[60]	64.61
3-Aminopropytriethoxysilane (SiNH_2_)	[61]	23.70

## 3. Experimental

### 3.1. General Information

All solvents and other chemicals (purity > 99.5%, Aldrich, Saint-Louis, MO, USA) were of analytical grade and used without further purification. Silica gel (E. Merck, Darmstadt, Germany) with particle size in the range of 70–230 mesh, median pore diameter 60 Å, was activated before use by heating it at 160 °C during 24 h. The silylating agent 3-aminopropyltrimethoxtsilane (Janssen Chimica, Geel, Belgium) was used without purification. All metal ions were determined by atomic adsorption measurements were performed by a Spectra Varian A.A. 400 spectrophotometer (Oujda, Morocco). The pH value was controlled by a pH 2006, J. P. Selecta s. a. (Barcelona, Span); Elemental analyses were performed by the Microanalysis Centre Service (CNRS, Lille, France). FT-IR spectra were obtained with a Perkin Elmer System 2000 instrument (Oujda, Morocco). SEM image were obtained on an FEI-Quanta 200 (Lille, France). The mass loss determinations were performed in 90:10 oxygen/nitrogen atmospheres on a Perkin Elmer Diamond TG/DTA, at a heating rate of 10 °C min^−1^ (Blois, France). The ^13^C-NMR spectrum of the solid state was obtained with a CP MAX CXP 300 MHz instrument (Lille, France). The specific area of the modified silica was determined by using the BET equation. The nitrogen adsorption-desorption was obtained by means of a Thermoquest Sorpsomatic 1990 analyzer (Lille, France), after the material had been purged in a stream of dry nitrogen. Molecular weights were determined on a JEOL JMS DX-300 Mass Spectrometer (CNRST, Rabat, Morocco).

### 3.2. Synthesis of 3-Aminopropylsilica (SiNH_2_)

The first stage in the preparation was the reaction between the silylating agent and silanol groups on the silica surface. Activated silica gel SiO_2_ (25 g) suspended in dried toluene (150 mL) was refluxed and mechanically stirred under nitrogen atmosphere for 2 h. To this suspension, aminopropyltrimethoxysilane (10 mL) was added dropwise and the mixture was kept under reflux for 24 h. The solid was filtered, washed with toluene and ethanol. It was then Soxhlet extracted with a 1:1 mixture of ethanol and dichloromethane for 12 h, to remove the silylating reagent residue. The immobilized silica gel, named SiNH_2_, was dried under vacuum at room temperature.

### 3.3. Synthesis of 1.5-Dimethyl-1H-pyrazole-3-carbaldehyde

(1.5-Dimethyl-1H-pyrazol-3-yl) methanol [62,63] (1.2 g, 9.52 mmol) was dissolved in 1,4-dioxane (100 mL). Activated manganese dioxide (21.88 g) was added to the solution and the suspension was stirred under reflux for 5 h. The reaction was monitored by TLC (alumina, CH_2_Cl_2_ as eluent). The hot suspension was filtered and the solid MnO_2_ was washed with boiling 1,4-dioxane. The solvent was removed on a rotary evaporator to give the expected product as a liquid (0.7 g, yield 59.23%). Rf = 47.51). ^1^H-NMR (CDCl_3_) δ ppm: 2.13 (s, 3H, C-CH_3_), 3.84 (s, 3H, N-CH_3_), 6.51 (s, 1H, Pz-H), 9.84 (s, 1H, C-H, aldehyde). ^13^C-NMR (CDCl_3_) δ ppm: 11.20(1C, C-CH_3_), 67.05 (1C, N-CH_3_), 105.19 (1C, CH_3_-C=C-), 140.88 (1C, C-CH_3_), 149.19 (1C, N=C-), 186.40 (1C, C-H, aldehyde). IR ν(C=O) = 1691cm^−1^. MS *m/z* = 124 (M^+^). 

### 3.4. Synthesis of ((1,5-Dimethyl-1H-pyrazol-3-yl)methylene)imine-Substituted Silica (SiNP)

For the synthesis of SiNP, a mixture of 3-aminopropylsilica (SiNH_2_, 5 g) and 1.5-dimethyl-1*H*-pyrazole-3-carbaldehyde (3 g) in dry ethanol (60 mL) was stirred at reflux for 8 h. After being filtered, the solid product was Soxhlet extracted with acetonitrile, methanol and dichloromethane for 12 h, respectively. The product was then dried under vacuum at 70 °C over 24 h.

### 3.5. Batch Experiments

The effects of solution pH and contact time on the sorption of metal ions were evaluated on batch method. A suspension of adsorbent (SiNP, 10 mg) in metal solution (10 mL) containing different concentrations of metal ions (243.01 mg/L for Pb(II), 102.93 mg/L for Cd(II), 75.63 mg/L for Cu(II) and 74.02 mg/L for Zn(II)), was mechanically stirred at room temperature for 1 min to 24 h at 25 °C and under various pH conditions. The mixture was then filtered off and the amount of metal ion in the filtrate solution was determined by atomic adsorption measurement using standard solutions for calibration. Analyses were performed in duplicate for each sample and only the mean data are reported. 

## 4. Conclusions

1.5-Dimethyl-1*H*-pyrazole-3-carbaldehyde was successfully bound on the silica surface after modification by 3-aminopyltrimethoxysilane. The structural, chemical and metal ion adsorption properties of this newly prepared sorbent was investigated. The adsorbent showed a high adsorption capacity towards Pb(II), Cd(II), Cu(II) and Zn(II) metal ions, with the best adsorption capacity of SiNP being 74.89 mg/g for Pb(II) ions. The new material was well characterized by elemental analysis, FT-IR spectra, ^13^C-NMR, nitrogen adsorption-desorption isotherm, BET surface area, B.J.H. pore sizes, thermogravimetry curves (TGA), and scanning electron microscopy (SEM). The new chelating surface exhibits good chemical and thermal stability. The sample was easily regenerated by soaking the sample in 6 N HCl for a few minutes (5–10 mL of 6 N HCl per g of support). The sorbent was regenerated five times, and showed no significant decrease in extraction percentage.
